# The sociability spectrum: evidence from reciprocal genetic copy number variations

**DOI:** 10.1186/s13229-020-00347-0

**Published:** 2020-06-16

**Authors:** Alejandro López-Tobón, Sebastiano Trattaro, Giuseppe Testa

**Affiliations:** 1 Laboratory of Stem Cell Epigenetics, IEO, European Institute of Oncology, IRCCS, Milan, Italy; 2 Department of Oncology and Hemato-oncology, Università degli studi di Milano, Milan, Italy; 3 Human Technopole, Via Cristina Belgioioso 171, Milan, Italy

**Keywords:** Sociability, Autism spectrum disorders, Hypersociability, 7q11.23, William-Beuren syndrome, 7dupASD, iPSCs

## Abstract

Sociability entails some of the most complex behaviors processed by the central nervous system. It includes the detection, integration, and interpretation of social cues and elaboration of context-specific responses that are quintessentially species-specific. There is an ever-growing accumulation of molecular associations to autism spectrum disorders (ASD), from causative genes to endophenotypes across multiple functional layers; these however, have rarely been put in context with the opposite manifestation featured in hypersociability syndromes. Genetic copy number variations (CNVs) allow to investigate the relationships between gene dosage and its corresponding phenotypes. In particular, CNVs of the 7q11.23 locus, which manifest diametrically opposite social behaviors, offer a privileged window to look into the molecular substrates underlying the developmental trajectories of the social brain. As by definition sociability is studied in humans postnatally, the developmental fluctuations causing social impairments have thus far remained a black box. Here, we review key evidence of molecular players involved at both ends of the sociability spectrum, focusing on genetic and functional associations of neuroendocrine regulators and synaptic transmission pathways. We then proceed to propose the existence of a molecular axis centered around the paradigmatic dosage imbalances at the 7q11.23 locus, regulating networks responsible for the development of social behavior in humans and highlight the key role that neurodevelopmental models from reprogrammed pluripotent cells will play for its understanding.

## Background

The evolution of human sociability and its complexity has been the subject of a long-standing debate, leading to a tense tug-of-war between schools of thought that favor either biological or cultural contributions as its main drivers [1]. Nevertheless, despite conflicting views regarding its causes, the importance of social functioning in the overall performance of an individual is unquestionable. Sociability is at the core of most behavioral tasks and arguably, to a large extent, essential to the biological *fitness* of individuals across species [2, 3].

Sociability has become a promiscuous term in recent years, describing numerous aspects of social interaction and functioning. In its overextended definition, sociability is an umbrella term that covers a wide spectrum of social features (e.g., social cognition, social behavior, social skills, social competence, social functioning) and the study of its aberrations is often associated to individuals with intellectual disability. However, sociability has no direct correlation to intellectual skills. Indeed, while the decreased intellectual ability is often comorbid with sociability aberrations (hypo or hypersociability), increased intellectual ability is not directly linked to sociability changes [4], indicating that healthy social behavior is dependent of sound intellectual ability, while social performance on itself bears no influence on intellectual skills.

The study of the distribution of sociability in the population shows that its manifestation constitutes a continuous variable fitting a normal (Gaussian) distribution, with most individuals falling in the middle of the range and some individuals exhibiting pathological/abnormal phenotypes, at each tail of the distribution [5]. Within this spectrum, the outlier groups make up two categories: 1) at the lower end, hyposociability, which encompasses psychopathic disorders, anxiety and autism spectrum disorders and 2) at the upper end, hypersociability, which includes the pathological need for social contact, emotional dependence on continuous social company and lack of social inhibition [6].

The high degree of specification of sociability definitions across different domains has made it difficult to find fitting animal models that reliably reproduce such features, usually requiring multiple extrapolations that are confounded by a myriad of underlying assumptions required for their interpretation [7, 8]. Thus, for the purpose of this review, we will equate sociability to *social cognition*, which is a more general term dedicated to humans, usually used in the diagnosis and which can be defined as the study of the ability to process, store and apply information about other people and social situations [9, 10].

In this work, we systematically review the neuroendocrine and genetic associations at both ends of the sociability spectrum; then, we proceed to highlight the insights offered by known reciprocal CNVs causing opposite sociability manifestations, with a particular emphasis on the role of dosage imbalances of the 7q11.23 locus.

## Neuroendocrine regulators of sociability

Most research regarding the molecular mechanisms regulating sociability has developed around the neuromodulatory functions of the neuroendocrine system, primarily centered on oxytocin (OXT) and arginine vasopressin (AVP) in the central nervous system (CNS). These closely related neuropeptides have been involved in broad areas of sociability such as affiliative behaviors (social bonding between individuals), pair-bonding (preference for contact with a familiar sexual partner), selective aggression towards unfamiliar conspecifics, biparental care, and socially regulated reproduction such as incest avoidance and aggressive behavior [11, 12]. OXT and AVP genes derive from the duplication of the vasocistin gene [13, 14] and encode for two nonapeptides with multiple actions [3]. Besides their systemic action, OXT and AVP acting in the central nervous system are responsible for the regulation of different aspects of social behavior. In the CNS, OXT is produced by the parvocellular neurons of the paraventricular nucleus, while central AVP is synthesized by the suprachiasmatic nucleus, bed nucleus of the stria terminalis, and medial amygdala [3, 15]. Studies in mice revealed that depletion of the AVP receptor 1b (*AVPR1B*) results in reduced aggressive behavior and social motivation [16, 17], while AVP itself promotes aggression or affiliation, depending on the social situation [18]. OXT is instead related to a general increase of sociability ranging from social memory to affiliating behavior, sexual or parenting, and aggression [19]. Oxytocin signaling is mediated by a G-protein coupled receptor (OXTR) [19, 20], and its knock out (KO) was associated with impairment in discriminating familiar from novel animals [19, 21]. In addition, it has been demonstrated that oxytocin activity in the amygdala diminishes fear behaviors through the activation of GABAergic interneurons [22, 23].

Genetic studies in patients with behavioral disorders have underscored a central role of OXT and AVP signaling pathways in human sociability. In particular, linkage disequilibrium studies have related the AVP receptor gene *AVPR1A* to autism [24]. Likewise, two microsatellites and two SNPs in the *AVPR1A* promoter have been associated with ASD [25–27]. Moreover, the chromosomic region 12q14, which includes the *AVPR1A* locus, was associated with autism through chromosome-wide haplotype analysis [28]. Similarly, polymorphisms in the OXT receptor, located in the 3p25.3 locus, have also been associated with autism [29–32] and deletion of the same chromosomic region causes intellectual disability, although its molecular etiology has been so far attributed only to the methyltransferase SETD5 [33]. Parallel observations showed that SNPs in CD38, a protein that regulates OXT release, have been associated with increased sociability and empathy [5, 34–36], suggesting a link between the modulation of OXT-mediated signaling and sociability.

The role of OXT pathway in favoring pro-social behavior and reducing anxiety has led to propose the administration of OXT as a potential treatment for autism, particularly through intranasal delivery [23, 37]. Remarkably, OXT has been used in several clinical trials for the treatment of Prader-Willi syndrome (PWS) [38–42]. However, although children and adults affected by PWS treated with intranasal OXT showed behavioral improvements in some cases, these observations have not been conclusive, mostly due to unfit statistical analyses and reduced patient cohort sizes in the available trials [43].

## Role of synaptic transmission in sociability

### Serotonin

One important mechanism by which OXT modulates social interactions is through its crosstalk with the serotoninergic neurotransmission, particularly by the interaction between oxytocin and serotonergic projections from the dorsal raphe to the nucleus accumbens [23]. Serotonin (5-hydroxytryptamine, 5-HT) is a neurotransmitter of complex multifaceted activity, produced in the CNS chiefly in the Raphe nuclei of the brainstem. Interestingly, multiple SNPs in the gene *GNAS*, encoding a G_αs_ subunit that couples with serotonin receptors 5HT4 and 5HT7, the AVP receptor 2 and dopamine receptors of the D1-like family, have been identified in a screening of ASD patients [44–47]. Genetic association studies also linked ASD to the enzyme that catalyzes the conversion of tryptophan into 5-hydroxytryptophan (5-HTP) in the brain, tryptophan-hydroxylase 2 (*TPH2*) [48–53]. In addition, hyperserotonemia in the peripheral blood is a biomarker of ASD [54], leading to the hypothesis that dysfunction of serotonin synapses caused by the alteration of its neuronal uptake or storage may have a direct behavioral impact [55]. In agreement, researchers found that the chromosomic region 17q11, harboring the gene encoding for the serotonin transporter SLC6A4, was strongly associated with ASD. Importantly, a SNP in this gene was also found to be associated with autism. Finally, serotonin and tryptophan were found at higher concentration in the peripheral blood and in the hippocampus of germ-free animals, which showed reduced anxiety in a sex-dependent manner [56–58]. These observations have led to propose the existence of a microbiota-gut-brain axis, defined by the associations between serotoninergic transmission in the central nervous system and gut microbiota, which could represent a therapeutic target for behavioral disorders [59].

### Dopamine

In parallel to the evidence pointing to a pivotal role for serotoninergic signaling in sociability regulation, recent studies increasingly suggest dopamine neurotransmission as an equally relevant component. Increased dopamine in the dorsal striatum causes sociability deficits and repetitive behaviors relevant to ASD, which were reversible by D1 receptor antagonists [60]. Interestingly, KO of the D2 dopaminergic receptors, coupled with Gαι subunit, is linked to the appearance of autistic-like behaviors [60]. Dopamine signaling in the mesocorticolimbic circuit, formed by neurons from the ventral tegmental area project to the prefrontal cortex and to the nucleus accumbens (which is part of the striatum), regulates reward and motivation-related behavior. Alteration of the dopaminergic signaling in the mesolimbic circuit results in reduced dopamine release in the prefrontal cortex and reduced response in the nucleus accumbens [61, 62]. These observations suggest the existence of a dopaminergic mesolimbic circuit that leads to persistent deficits in social interaction and communication in ASD [61, 63].

### Endocannabinoids

Endocannabinoids also regulate social behavior through striatum circuits and habitual/compulsive motor routines by feedback loop-inhibition of glutamate release by neurons projecting from brain regions that serve emotional cognitive sensory and motor functions on the medium spiny neurons residing in this brain region [64–69]. This function is mediated by the signaling of the cannabinoid-1 receptor activated upon binding of the 2-arachidonoyl glycerol (2-AG), the most abundant endocannabinoid in the brain [70–72]. Given the evidence pointing to an increased hyper-glutamatergic activation and its link to diacylglycerol metabolism as one of the determinants of ASD [73, 74], researchers have concentrated their work in *Dagla*, a gene encoding for the diacylglycerol lipase involved in 2-AG biosynthesis. Conditional KO of *Dagla* in different regions of the striatum affected the sociability (dorsal striatum dependent) and caused repetitive behaviors (ventral striatum dependent). Intriguingly, a paternal-inherited deletion disrupting the DAGLA gene was found in a patient with ASD, although data show that this genetic lesion is not fully penetrant [64].

### Glutamate

**Fig. 1:**
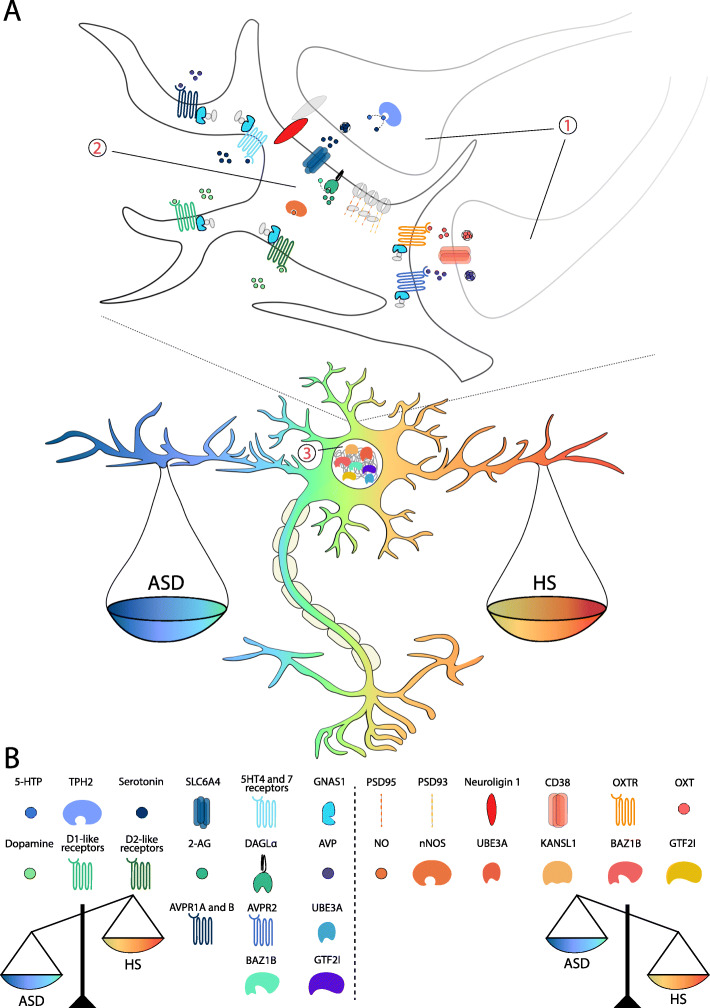
The sociability spectrum: a molecular overview. (**a**) Graphical representation of the main neuroendocrine and epigenetic pathways involved in the molecular regulation of sociability. The pre- (1) and the post-synapsis (2) as well as the epigenetic and transcriptional regulation of gene expressions (3) define social behavior, which can range in a wide spectrum of normal conditions. When the misfunction of genes involved in these neuronal functions takes place, the two extremes of the spectrum, autism spectrum disorders (ASD) and hypersociability (HS), manifest. (**b**) Graphical legend of all the molecular components associated with ASD and HS described in this review

Most of the work regarding sociability has focused only on one side of the spectrum, namely the dysfunctions in social behavior leading to autism. Interestingly, a considerable amount of molecular evidence has pointed to the involvement of the glutamatergic synapses in the regulation of “hypersocial” behaviors. Indeed, throughout the years, different single genes have been linked to hypersociability, including important regulators of the pre- and post-synapsis (Fig. 1). A prime example is *Dlg4*, which encodes for the post-synaptic density protein PSD95, a pivotal protein of the postsynaptic compartment involved in the stabilization of the NMDA receptors by direct biding and anchoring [5, 75]. *Dlg4* null mice displayed higher interaction with unfamiliar animals in social recognition and novelty tests. Interestingly, heterozygous mice for *Dlg4* had only hypersociability, as opposed to deletions of its homologue *Dlg2* (encoding for PSD93), which displayed increased sociality only in the homozygous KO situation [76, 77]. A similar phenotype was observed with Neuregulin-1 (*Nrg1*) haploinsufficiency, a component of an EGF-like signaling module that interacts with ERBB receptors and is crucial for the regulation of cell-cell communication, neuronal migration, and glutamate signaling [78, 79]. Finally, indirect glutamatergic synapse modulators have shown likewise to have behavioral consequences. One example is the neuronal nitric oxide synthase (nNOS), an enzyme responsible for the synthesis of nitric oxide in the postsynaptic terminal, which acts through retrograde signaling to activate soluble guanylyl cyclase in the presynaptic terminal, thus regulating neurotransmitter release [80, 81]. Disruption of nNOS activity causes improvement or worsening of sociability in the presence of familiar or unfamiliar animals, respectively [82].

## Reciprocal genetic dosage imbalances and its sociability manifestations

Studying neurodevelopmental disorders (NDDs) of defined genetic origin displaying highly penetrant aberrant sociability phenotypes has been instrumental in identifying candidate genes underlying neurodevelopmental mechanisms of sociability. However, one of the biggest challenges when understanding the genetic etiology of behavioral disorders and, in particular, ASD, is the remarkable heterogeneity of their genetic associations and wide range of phenotypic manifestations and comorbidities, which has the historically complicated diagnosis and hindered the development of therapies [83]. Genetic copy number variations (CNVs) account for about 10% of all behavioral disorders and intellectual disability of genetic origin, typically featuring paternal and age biases [84, 85]. The uncovering of the molecular mechanisms underlying the social manifestations in humans has been hampered by the lack of convergence between the genetic lesions and phenotypic manifestations in animal models [86]. Therefore, the onset of cell reprogramming for obtaining induced pluripotent stem cells (iPSCs) introduced a shift of paradigm that is allowing the interrogation of these molecular pathways on a human genetic background in an ever-growing number of NDDs (Table 1). For instance, in a landmark study, researchers derived iPSCs from patients affected by Down Syndrome (DS) and differentiated them into neural progenitors, whose maturation was followed in vivo after transplantation into mice brains. By using single-cell-resolution intravital microscopy they were able to find that dendritic spines and synaptic boutons of DS-derived neurons were aberrantly more stable than in controls, all of which hints to dysfunction of synaptic plasticity that may ultimately reverberate on sociability [106]. More recently, the use of iPSC-derived neurons from Kleefstra Syndrome patients helped to uncover a specific anomalous pattern of excitatory network activity that could be rescued by the administration of NMDARs pharmacological inhibitors, bearing breakthrough potential for therapeutic application [107].

**Table 1:** List of representative sociability-related CNVs with reported iPSCs-derived neural models

Disease	Locus	Type of CNV	Phenotype	Refs
	16p11.2	Deletion/Duplication	ASD	[87–89]
	15q11.2	Deletion	ASD	[90–92]
Phelan-McDermid Syndrome	22q13.3	Deletion	ASD	[93–96]
	Xp22	Deletion	ASD	[97, 98]
	2p16.3	Deletion	ASD	[99]
	15q13.3	Deletion	ASD	[100]
Kleefstra syndrome	9q34.3	Deletion	ASD	[101–103]
Down Syndrome	Chromosome 21 trisomy	Duplication	Hypersociability	[104–106]

The application of iPSC-derived models to study reciprocal (or mirrored) CNVs with opposite behavioral impact represents a particularly fertile field to interrogate the effect of gene-dosage imbalances in social behavior [108]. This type of mirrored modifications entailing opposite sociability features is however extremely rare, making up for just a short list of coupled disorders (Table 2). Among these disorders, only 7q11.23 and 15q11.13 represent reciprocal genetic dosages with “truly” opposite hyper and hyposociability manifestations, whereas the psychiatric features of 1q21.1 microdeletion and microduplication exemplify a proposed model in which autism and schizophrenia represent two opposite extremes of a spectrum reflecting the under-development or over-development of the social brain [123]. Moreover, the 15q11.13 phenotypic manifestations derive from changes in gene dosage that are not only exclusively caused by microdeletion or microduplication of the locus but also by uniparental imprinting disomy leading to imbalanced allele silencing, [124–126]. Thus, 7q11.23-related syndromes constitute the only known pair of reciprocal CNVs with highly penetrant opposite sociability manifestations, which make them uniquely relevant for the unbiased interrogation of dosage effects.

**Table 2:** Reciprocal CNVs associated with mirrored behavioral phenotypes

Disease	Locus	Type of mutation CNV	Behavioral phenotype	Comments	Refs
	1q21.1	Deletion	ASD		[109, 110]
	1q21.1	Duplication	Psychosis/schizophrenia		[111]
Williams-Beuren syndrome	7q11.23	Deletion	Hypersociability	Language skill preserved	[112, 113]
7dupASD	7q11.23	Duplication	ASD		[114, 115]
Angelman syndrome*	15q11-q13	Deletion (paternal)	Hypersociability	Deletion paternal allele ~75% cases LoF mutation UBE3A ~ 11 % Language skills impaired	[116–118]
Prader-Willi syndrome*	15q11-q13	Deletion (maternal)	ASD	Deletion maternal allele ~ 70% cases Maternal uniparental dysomy ~ 20 %	[119–121]
15q11-q13 microduplication syndrome	15q11-q13	Duplication	ASD		[122]

*Syndromes related not only to direct CNV but also to changes in gene dosage due to gene imprinting

### 1q21.1

The recurrent distal 1.35-Mb 1q21.1 microdeletion is an inherited autosomal dominant aberration leading to a series of symptoms with no clear syndromic association [110]. Between 18% and 50% of deletions occur de novo. The microdeletion can be inherited from either parent who can be carriers displaying a less severe phenotype [127]. Its phenotypic manifestations are quite variable, including individuals with no obvious clinical features while others display variable signs including microcephaly (50%), mild intellectual disability (30%), and mildly dysmorphic facial features and eye abnormalities (26%). The most frequent psychiatric and behavioral abnormalities are autistic features, followed by attention deficit hyperactivity disorder and sleep disturbances [110]. The 1q21.1 microduplication is instead associated with developmental delay, congenital anomalies, and macrocephaly in children [128]. Its psychiatric manifestations are likewise variable, including in many cases ASD; however, the high incidence of schizophrenia and psychosis, typically absent in deleted patients, led us to include it as an example of opposite behavioral manifestations (Table 2) [123].

The 1q21.1 variable CNV typically encompasses 15 genes (*PDE4DIP, HYDIN2, PRKAB2, PDIA3P, FMO5, CHD1L, BCL9, ACP6, GJA5, GJA8, NBPF10, GPR89B, GPR89C, PDZK1P1,* and *NBPF11*) and the molecular mechanisms underlying their pathogenic impact are poorly characterized. A gene expression association study using the peripheric blood of 1q21.1 microduplication patients found a significant dysregulation of language associated genes, including *CDH1L* and *ROBO1*, both highly upregulated, whereas, *TLE3*, a target of *FOXP2* was significantly downregulated [129]. These changes could potentially explain language and particularly speech dysfunction. However, in the absence of mechanistic links and confirmation with additional probands, these associations remain speculative. A dedicated mouse model carrying a synthetic 1q21.1 microduplication found schizophrenia-like behaviors as well as increased hyperactivity in response to amphetamine challenge. A battery of inhibitors testing showed a direct dependence of D1/D2 dopaminergic receptors, constituting the first molecular link to the behavioral impact of 1q21.1 CNV [129].

### 15q11-q13

Variations in gene expression dosage at the 15q11-13 locus cause a group of related syndromes, Prader-Willi syndrome (PWS), Angelman syndrome (AS), and 15q11-13 microduplication syndrome [111, 116, 130]. PWS is caused by a lack of the paternally derived imprinting of the chromosomic region 15q11-13, either through paternal deletion or maternal uniparental disomy and is characterized, among other features, by mild to moderate levels of intellectual disability, compulsive behaviors, ASD and increased risks of morbid obesity [117, 118]. AS, the counterpart of PWS syndrome, is caused by maternal deletion of chromosome 15q11-13 and in particular of the gene coding for E3 ubiquitin ligase 3A (*UBE3A*). Among its typical features are found microcephaly, severe intellectual deficit, speech impairment, whereas from a behavioral point of view, patients display general happiness and frequent smiling and laughing as well as hyperactivity [119, 131, 132]. These sets of behaviors have been grouped as hypersociability for their proven association to increased motivation to interact with others in social situations [120].

The molecular mechanisms behind the sociability disruption in 15q11-13-related syndromes have been widely studied and chiefly associated with *UBE3A* [121, 133], thought to be the main responsible for the increased risk of ASD in PWS patients [134, 135]. Transgenic mice carrying an *Ube3a* duplication showed a dose-dependency of its gene product to sociability manifestations, in particular fact, mice with maternally-inherited *Ube3a* deletion displayed a prolonged preference interaction with social stimuli in the three-chamber social approach task [121]. Mechanistic dissection showed that the accumulation of UBE3A in the nucleus downregulates the glutamatergic synapse organizer CBLN1, which is needed for sociability in mice, through the regulation of the activity of VGLUT2-expressing neurons in the ventral tegmental area (VTA) [136]. More recently, the use of AS patient-derived neurons and brain organoids allowed a first demonstration of a direct role of UBE3A in the suppression of neuronal hyperexcitability by inducing the degradation of calcium and voltage-dependent big potassium (BK) channels, thus avoiding heterochronic network synchronization, which is a primary cause of epileptic seizures [137].

Similar phenotypes to AS have been observed in Koolen-De Vries syndrome (KdeVs), which is caused by haploinsufficiency of the *KANSL1* gene [138, 139]. In this case, however, *Kansl1* haploinsufficient mice did not recapitulate the increased sociability [140]. Likewise, Down syndrome (DS), caused by trisomy of chromosome 21, displays several traits of hypersociability, including good social skills and affectionate interactions, while showing a lower prevalence of aggression and antisocial behavior, although a defined gene candidate underlying these features is yet to be identified [4, 104, 105, 141].

## 7q11.23 CNV syndromes as paradigmatic examples

Copy number variations at the 7q11.23 locus cause a pair of paradigmatic syndromes (deletion, Williams-Beuren syndrome, WBS and duplication, 7dupASD) entailing an almost full-penetrance of opposite social manifestations, with 7dupASD receiving an ASD diagnosis in over 90% of the cases and WBS manifesting a wider spectrum of hypersociability-related features compared to other hypersociability syndromes, including an unusual combination of intellectual disability with preservation of language skills [142, 143]. WBS and 7dupASD are autosomal dominant disorders caused by genomic rearrangements due to large region-specific low-copy repeat elements (LCR) and Alu transposable elements that may lead to non-allelic homologous recombination if not correctly aligned during meiosis [112, 113, 144]. Their reported incidence in the population is about 1/10000 for WBS and 1/20000 for 7dupASD.

The deletion/duplication of the Williams-Beuren syndrome critical region (WBSCR) leads to hemizygosity/hemiduplication of 25-28 genes that account for their phenotypic manifestations [145, 146]. Among others, the WBSCR contains genes encoding transcriptional regulators such as *GTF2I*, *GTF2IRD1*, *BAZ1B*, *MLXIPL*, or signaling molecules *FZD9*, *TBL2*, *LIMK1* [145]. Following a classification by Golzius and Katsanis [108], these couple of syndromes belong to the most complex type of CNV or “complex cis-epistatic” model, in which phenotypes are the result of the simultaneous dosage imbalances of numerous genes within the CNV, some of which drive specific endophenotypes and some of which exhibit complex additive and/or multiplicative relationships.

WBS patients present different phenotypes with different degrees of expressivity, including supravalvular aortic stenosis, hypercalcemia, persistent growth failure, facial dysmorphisms, mental retardation, and hypersociability, but often they do not show all these defects together. Indeed, prior to the characterization of a patient showing all phenotypes, WBS was considered two different disorders [114, 147]. To date, FISH and microsatellite marker analysis represent the standard laboratory tests for unequivocal diagnosis [113, 144, 147]. The first gene mapped in the WBSCR that was directly linked to a phenotype was the gene coding for elastin (*ELN*), which causes the cardiovascular and connective tissue phenotype of the disease (i.e., SVAS) [144]. WBS patients have delays in the acquisition of early motor and language skills and show mild-to-moderate intellectual disability in adulthood (IQ from 50 to 60) [147]. Likewise, WBS patients display defects in visuospatial and visuomotor skills (the ability to spatially relate objects), which has been related to the hypersociability phenotype due to the atypical evaluation of facial trustworthiness [148, 149]. Despite this, they display relative strengths in facial recognition and interpersonal skills, supported by their proficient language [150, 151]. Interestingly, WBS patients usually enjoy music, but very often develop sensitivity to certain noises (selective hyperacusis) [145]. The hypersociability characteristic of patients with WBS is associated with excessive worry and fears; indeed, more than 80% of adults with WBS show anxiety (but not social anxiety), preoccupations or obsessions, irritability, and distractibility [147].

Opposite to WBS, 7dupASD is characterized by cognitive abnormalities, such as language impairment and deficits of social interaction, epilepsy, anxiety, and mild dimorphisms [150, 152]. 7dupASD patients show both similar and opposite features compared to WBS patients [143, 146]. It is characterized by various symptoms ranging from severe speech impairment to classical autistic disorders and craniofacial dysmorphisms [143].

The characterization of WBS patients with atypical breakpoints in the WBSCR allowed the study of the specific genes of the region, partially elucidating their contribution to the cognitive, behavioral, and neural phenotype seen in WBS [113, 151]. One conspicuous case emerged from atypical deletions has been the phenotype shown by sparing genes from the TFII-I family present in this region (*GTF2I*, *GTF2IRD1*, and *GTF2IRD2*) [150, 151, 153–156]. This gene family shares a number of similar intragenic repeats coding for helix-loop-helix structures required for DNA binding and is probably the result of intragenic duplications that occurred during evolution [144]. Phylogenetic reconstruction of GTF2I, GTF2IRD1, and GTF2IRD2 proteins demonstrates that GTF2I and GTF2IRD1 had a common ancestor in early vertebrates. These two genes are found in all land vertebrates and are located close to each other with the same orientation suggesting an ancient duplication. A second duplication, this time with inversion, led to the origin of *GTF2IRD2*. The final duplicative re-arrangement of the 7q11.23 locus generated *GTF2IRD2B* during late primate evolution and included a second inversion event which so far has been observed only in the human genome [157].

In mice, GTF2I regulates the expression of the *DLX* homeobox gene involved in the differentiation and migration of GABA-expressing neurons in the forebrain, suggesting that the dosage of GTF2I could alter the excitation/inhibition balance [156], In agreement with multiple evidence suggesting an imbalance excitation/inhibition ratio of cortical neurons as an underlying substrate of sociability network development [115, 158].

Comparative studies addressing the mechanisms that drive the heightened propensity of dogs to initiate social contact, when compared with human socialized gray wolves, explained this behavior as a type of behavioral neoteny, the retention of juvenile features in the adult [159], which is on itself potentially the result of transcriptional neoteny in the brain [160]. Interestingly, a genome-wide association of SNP in dogs from 85 breeds vs 92 gray wolves identified a top-ranking outlier locus located within the polymorphic *WBSCR17* gene, which is typically deleted in WBS. A follow-up study found that a 5 Mb genomic region around the Williams critical region was under positive selection in domestic dog breeds and that hypersociability is a core element of domestication that distinguishes dogs from wolves [159]. Interestingly, this divergence seems to be directly linked to structural variants in *GTF2I* and *GTF2IRD1*, placing *GTF2I* and its surrounding locus at the core of targets with likely direct contribution to the development of brain networks regulating sociability.

In humans, the role of the TFII-I family in determining the hypersociability phenotype has started to be elucidated. Patients carrying atypical deletions sparing only *GTF2I* do not show hypersociability, but only visuospatial construction deficits and craniofacial features [145], further emphasizing its role in sociability development. Instead, *GTF2IRD1* has been associated with the visuospatial abilities [113, 144, 145, 151]. Moreover, a GTF2I deficit was found in the hippocampus of WBS patients, supporting its contribution to the characteristic spatial cognition deficit of those individuals [161]. Importantly, GTF2I interacts with the serotonin receptor 3A and mutation in *GTF2I* has been associated with alteration in serotonin currents in the prefrontal cortex [162]. These findings are in line with the hypothesis of GTF2I at the center of the hypersociability phenotype observed in WBS, also in agreement with the role of the serotonin system in regulating social cognition and anxiety.

### Use of patient-derived models

While animal models have proven instrumental in uncovering many of the molecular underpinnings of the development of the social brain, some of its human-specific aspects, chiefly those linked to the significantly more complex cortical development, will require models that factor the human genetic background into this mix, such as brain organoids, which have been shown to recapitulate unique aspects of human cortical development 10.1038/nrn.2017.10710.1038/nature1251710.1016/j.cell.2019.01.01710.1016/j.stemcr.2019.09.00510.1016/j.stem.2016.11.017. An important hurdle is the recurrent failure in recapitulating many phenotypes in a hemizygous condition [163], unmasking obvious differences in susceptibility and phenotype penetrance due to genetic background.

Work done in our group through transcriptional analysis of human-induced pluripotent stem cell (iPSCs) derived from WSB and 7dupASD patients revealed that many of the biological processes predictive of the disease manifestation (i.e., related to brain development) are found altered already at the pluripotent state in the two conditions [146]. About 10–20% of this transcriptional deregulation was attributed exclusively to GTF2I and this dysregulation was propagated into disease-relevant lineages, including neural crest and neural progenitors [146]. Interestingly, we found that GTF2I not only acts as a transcriptional activator but also is responsible for gene repression through its interaction with LSD1 and HDAC2. These observations provided the first molecular evidence of early transcriptional dysregulation as a potential mechanism explaining the gene dosage imbalances of GTF2I and sociability aberrations [149].

Another crucial gene of the WBSCR is *BAZ1B*. We recently demonstrated that BAZ1B is the master regulator of the modern human face, on the basis of a functional molecular dissection of its dosage imbalance in patient-derived neural crest stem cells (NCSCs) [164]. We found that BAZ1B regulates the developing NCSCs derived from patient iPSCs, starting from its earliest migratory stages by downregulating well-established critical regulators of NCSCs migration and maintenance, confirming that its dosage imbalances, characteristic of WBS and 7dupASD, alter NCSCs migration. Interestingly, the gracilization of the cranium has been strongly associated with the “self-domestication hypothesis”, which proposes that social behavior co-evolved with specific craniofacial features through natural selection of traits that favored increased in-group prosociality over aggression in the *H. sapiens* lineage [165–167]. In WBS, the lower-mid face morphology sharply departs from the anatomically modern human one with traits that can be reconducted to a further gracilization of the cranium, which may be related to the hypersociability phenotype characteristic of this syndrome [164].

## Conclusions

**Fig. 2:**
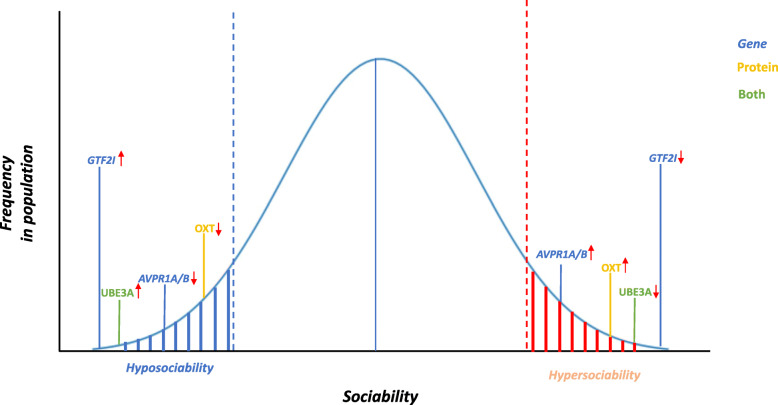
Summary of genes and proteins with reciprocal actions in the sociability spectrum in humans. Positioning of molecular players for which evidence is available of opposite actions at both ends of the sociability spectrum in terms of gene expression (blue) and protein levels (yellow), or both (green) and the respective directionality of their dosage (arrows)

Sociability is a phenotypic domain that reaches unparalleled complexity in humans. The study of behavioral disorders and its genetic causes has allowed to define a complex landscape of genes and molecules that play pivotal roles at both ends of the sociability spectrum. Considering that by definition gene dosage/function defects in these disorders are present from early development, an open question is whether their role is exclusively influencing developmental circuits or whether they may be modulating the function of the mature social brain. A particularly relevant subset of genes and proteins involved in several of these syndromes are those whose dosage seems to be directly linked to a sociability outcome (Fig. 2), indicating their potential key role in defining the circuits that regulate social behavior in humans. Since the generation of animal models has proven often disappointing in recapitulating social phenotypes, it becomes salient the importance to maintain a human genetic background. To further unveil the developmental trajectories and specific cell populations affected by specific gene dosages, will require the genetic engineering of human pluripotent cell lines with multiple allelic series of endogenous expression and their differentiation into nerve cells using more comprehensive models such as brain organoids, which will allow to simultaneously address the uniqueness of human brain development within the context of the human-specific genetic background.

## Data Availability

Not applicable.

## Abbreviations

ASD: Autism spectrum disorder
CNV: Copy number variation
OXT: Oxytocin
OXTR: Oxytocin receptor
AVP: Vasopressin
CNS: Central nervous system
PWS: Prader-Willy syndrome
5-HT: 5-hydroxytryptamine
5-HTP: 5-hydroxytryptophan
TPH2: Tryptophan-hydroxylase 2
NDD: Neurodevelopmental disorder
AS: Angelman syndrome
KdeVS: Koolen-De Vries syndrome
DS: Down syndrome
WBS: Williams-Beuren syndrome
LCR: Low-copy repeat elements
7dupASD: 7q11.23 duplication autism spectrum disorder
WBSCR: Williams-Beuren Critical Region
NCSC: Neural crest stem cells
HS: Hypersociability
iPSC: Induced pluripotent stem cells
KO: knock-out
